# It’s Getting Hot in Here: A Rare Case of Heat Stroke in a Young Male

**DOI:** 10.7759/cureus.3724

**Published:** 2018-12-12

**Authors:** Oriana Ramirez, Yury Malyshev, Sonu Sahni

**Affiliations:** 1 Department of Internal Medicine, Brookdale University Hospital Medical Center, New York, USA

**Keywords:** heat stroke, cardiac arrest, altered mental status, critical care medicine, hyperthermia

## Abstract

Heat stroke is a severe acute illness characterized by a core temperature greater than 40°C (104°F) and central nervous system manifestations, such as delirium, convulsions, or coma, resulting from exposure to environmental heat or strenuous physical activity. Early recognition and treatment including aggressive cooling and management of life-threatening systemic complications, such as cardiac arrest, rhabdomyolysis and acute renal failure, are essential to reduce morbidity and mortality. Herein we describe a case of heat stroke in a 23-year-old male who suffered cardiac arrest in which prompt initiation of cooling measures prevented permanent neurological sequelae, provided swift neurological recovery and resolution of impending multi-organ dysfunction syndrome.

## Introduction

Heat stroke is an often life-threatening condition which is defined as an elevated core body temperature above 40°C (104°F) with central nervous system (CNS) involvement that may manifest as confusion, delirium, seizure-like activity or coma [1]. The mainstay of therapy remains adequate cooling of the body temperature and aggressive fluid resuscitation. However, heat stroke still remains often fatal and resulting in permanent neurologic damage in those who survive [2]. The elderly are the most commonly affected population, due to presence of comorbidities and impaired internal thermoregulation. The main risk factors for developing heat stroke remain heat waves, excessive humidity, alcohol consumption and intense physical activity [3]. Diagnosis of heat stroke is complicated by the lack of a specific diagnostic testing, vague presentation and non-specific lab abnormalities. Herein we describe a rare case of heat stroke in a young male with a complicated course that presented a clinical challenge. The information presented in this report aims to create awareness as clinical outcomes depend on early recognition and management.

## Case presentation

**Figure 1:**
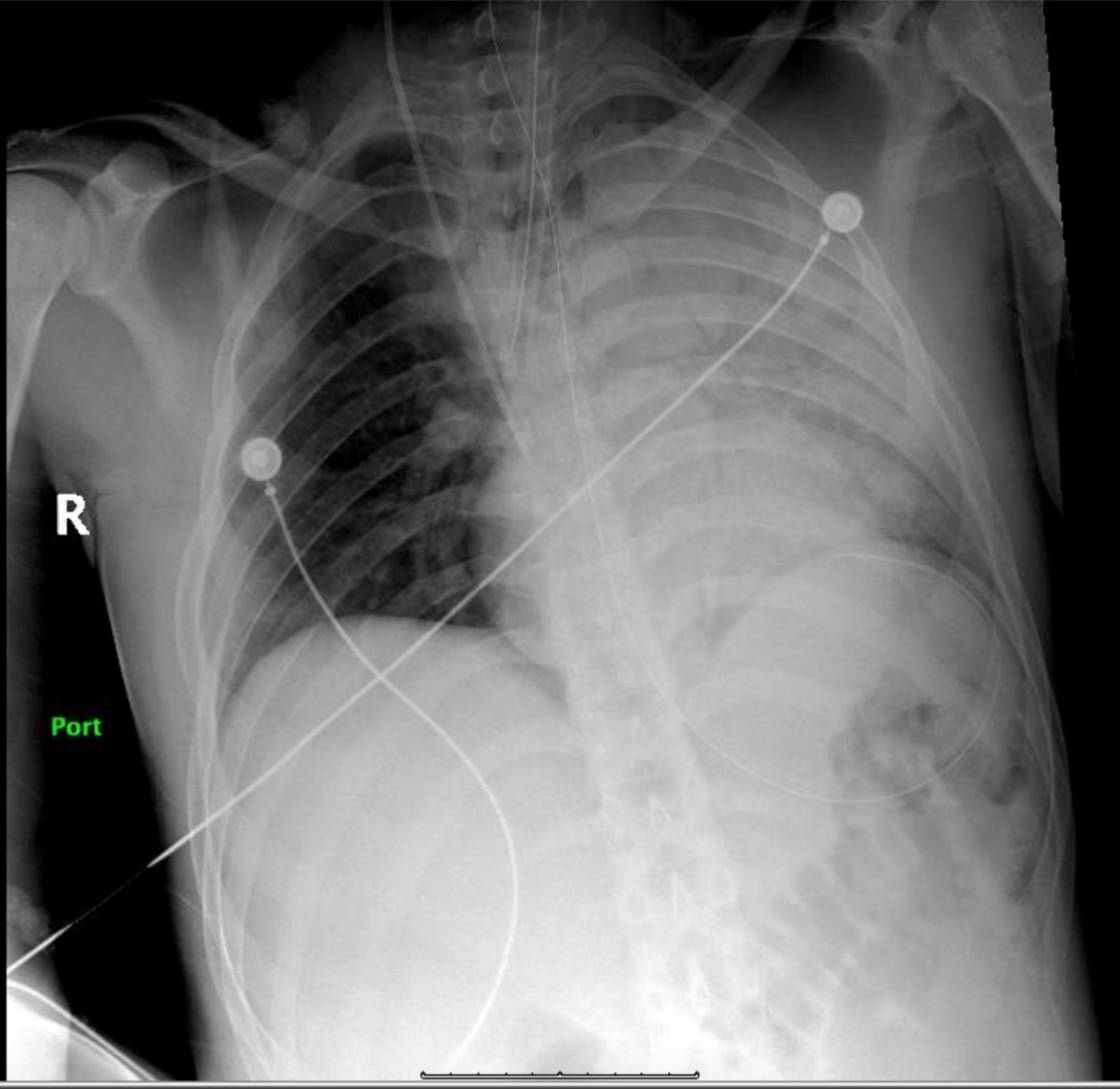
Chest X-ray (CXR) demonstrating a dense opacity noted throughout the left lung due to pneumonia and/or atelectasis.

A 23-year-old male with no prior medical history was found unconscious in a construction building. Minutes prior to the incident the patient was noticed to be walking and behaving erratically, drinking excessive amounts of fluids. A friend who accompanied the patient specified that the patient was not engaged in overly exertional work and that he was wearing light clothing. Further questioning revealed no history of substance abuse and no previous history of heat-related illnesses. On arrival to the emergency department (ED) the patient was in obvious distress, normotensive (BP 102/64 mmHg), tachycardic (HR 159 bpm), tachypneic at 42 breaths per minute and febrile on rectal temperature (43.3°C/109.9°F). Initial electrocardiogram (EKG) showed sinus tachycardia. Initial labs showed electrolyte disturbances, abnormal transaminase and acute kidney injury. In addition, elevated troponin and creatine kinase were observed. Laboratory values have been shown in Table 1. Urinalysis was negative for infection. Cooling protocol was initiated with use of ice packs when the patient suddenly became unresponsive. The patient was intubated to secure the airway and a few minutes thereafter he suffered cardiac arrest. Advanced cardiovascular life support was initiated and the code was run for nine minutes. The patient received three doses of epinephrine, three doses of bicarbonate and two doses of calcium gluconate. Initially the rhythm was reported as pulseless electrical activity (PEA) followed by ventricular fibrillation; after which the patient was defibrillated achieving return of spontaneous circulation (ROSC). Broad spectrum antibiotics including coverage for meningitis and aspiration pneumonia (vancomycin, ampicillin, ceftriaxone and metronidazole) were initiated. Lumbar puncture was unremarkable. Chest radiograph after the cardiac arrest showed left side infiltrates (Figure 1). Hypothermia protocol was started and the patient was transferred to medical intensive care unit (MICU).

**Table 1:** Laboratory values from admission to the end of intensive care unit (ICU) course.​​ MICU: Medical intensive care unit; AST: Aspartate transaminase; ALT: Alanine transaminase.

Laboratory Test (Normal Range)	Initial	End of MICU course
Hemoglobin g/dL (11.4-15.5 g/dL)	12.9	11.6
Hematocrit % (37.0-43.7%)	37.1	32.8
White Blood Cells 10^9^/L (4.5-10.2 x 10^9^/L)	7.8	14.2
Platelets 10^9^/L (180-401 x 10^9^/L)	58	871
Blood Urea Nitrogen mg/dL (7.0-17.0 mg/dL)	33	32
Creatinine mg/dL (0.52-1.04 mg/dL)	1.91	0.76
Sodium mEq/L (133-145 mEq/L)	129	138
Potassium mEq/L (3.5-5.1 mEq/L)	2.9	4.3
AST/ALT U/L (14-36 U/L / 9-52 U/L)	203/140	65/124
Lactate mmol/L (0.70-2.10 mmol/L)	8.5	1.4
Troponin ng/mL (0.00-0.034 ng/mL)	1.99	0.049
Creatine Kinase U/L (55-170 U/L)	>3200	336

**Figure 2:**
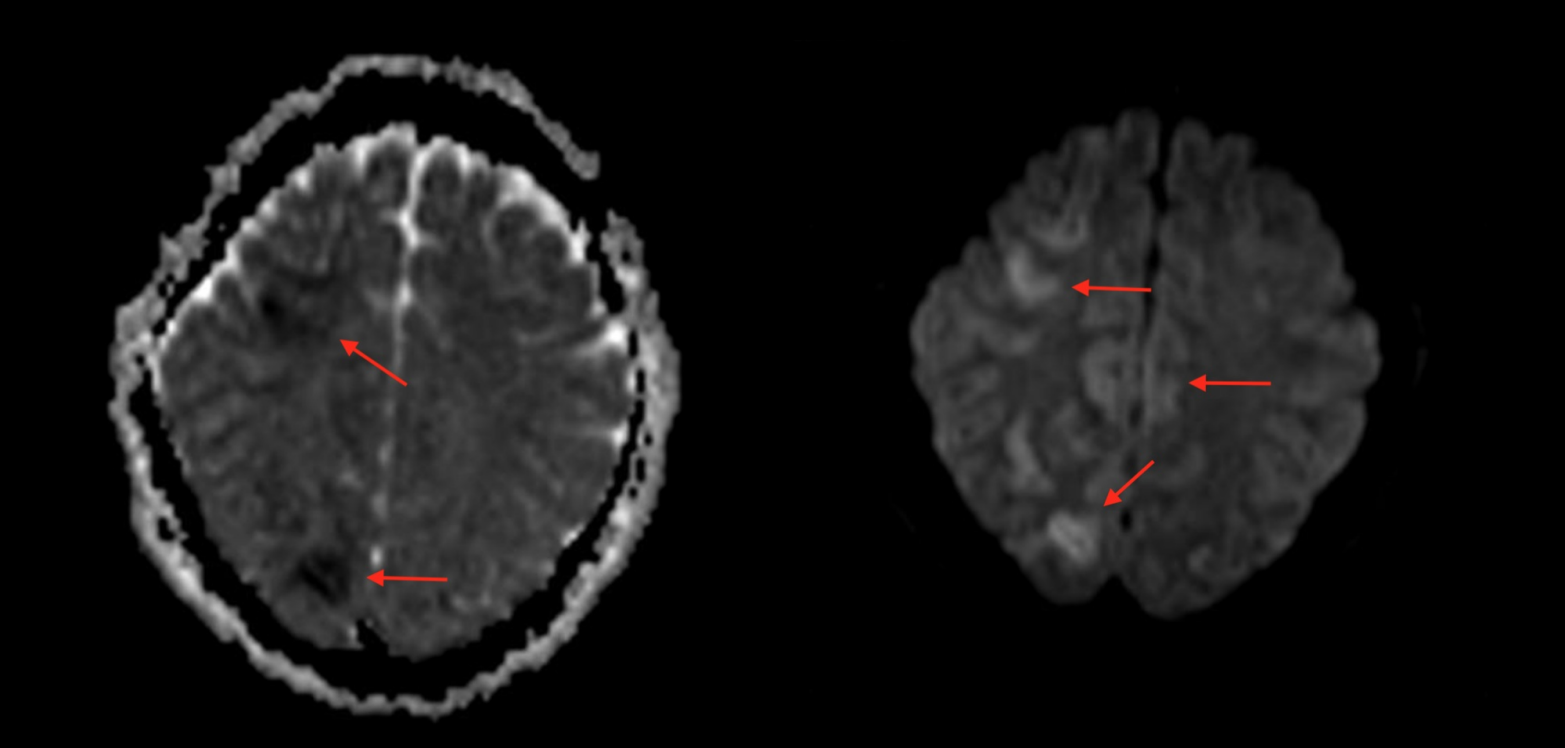
Magnetic resonance imaging (MRI) of the brain showing patchy areas of restricted diffusion in both cerebral hemispheres, more extensive on the right suspicious for acute infarction. Left - MRI EP2D-DIFF 3-Scan Trace image; right - MRI EP2D-DIFF 3-Scan Trace ADC image. ADC: Apparent diffusion coefficient

Due to worsening infiltrates on Chest X-ray and PO2/FiO2 < 300, acute respiratory distress syndrome (ARDS) protocol was initiated. Ceftriaxone and metronidazole were continued, along with transcutaneous pacing due to sinus bradycardia. After rewarming, 48 hours later, physical examination showed a Glasgow Coma Scale score of seven with preserved brain stem function. Subsequently, the patient was witnessed to have several episodes of clonic seizures. Magnetic resonance imaging (MRI) of the brain showed patchy areas of restricted diffusion of both cerebral hemispheres, more extensive in the right in a watershed distribution (Figure 2). Subsequent electroencephalogram revealed severe diffuse metabolic encephalopathy. Urine toxicology screen was positive for benzodiazepines that were administered during the admission. Seven days after admission the patient started showing signs of neurological improvement. He demonstrated left side upper and lower extremity hemiparesis and full range of activity on the right evidenced by following simple commands. The patient was extubated with no complications and nasogastric tube feeding was continued but due to patient agitation, percutaneous endoscopic gastrostomy (PEG) tube was placed to provide adequate nutrition. The patient showed progressive neurological improvement, following more commands with the right-side extremities, receptive language skills with hands and improved swallowing movements. Before discharge the right side of the body was completely functional. Physical therapy was continued as outpatient.

## Discussion

Herein we describe a unique case of heat stroke in young male complicated by cardiac arrest. Heat stroke is an infrequent diagnosis in countries with temperate climate, however, there has been an increased frequency of heat waves which is associated with heat-related deaths [4]. Heat stroke is a life-threatening condition characterized by hyperpyrexia (elevated core body temperature exceeding 40°C) which potentiates a systemic inflammatory response leading to multiorgan dysfunction syndrome (MODS) in which encephalopathy predominates [1, 5]. Despite adequate lowering of the body temperature and aggressive treatment, heat stroke is often fatal with a hospital mortality reported to be up to 65% [6]. Those who do survive often sustain permanent neurologic damage [7]. Data of incidence of heat stroke are imprecise as it is often underdiagnosed and the reporting of heat-related death varies [8, 9]. During 2006–2010, about 2,000 U.S. residents died each year from weather-related causes of death of which 31% were attributed to exposure to excessive natural heat [10].

Two main categories of heat stroke have been described in the literature based on pathoetiology. The first is from high environmental temperature (referred to as classic or non-exertional heat stroke) and the second from strenuous physical activity (referred as exertional heat stroke) [5]. There are many factors that may contribute to heat stroke in addition to environmental conditions such as, lack of acclimatization, wearing tight or excessive clothing, obesity, extremes of age, concurrent comorbidities such as heart disease or diabetes and states of dehydration. Excessive alcohol use, diuretics, beta-blockers, anticholinergics, antihistamines, illicit drugs like amphetamines, MDMA and cocaine and activities involving prolonged exertion with poor fluid intake may also put individuals at risk [5]. Most commonly in the younger population, as in our case, there is a component of physical exertion involved which may be exacerbated by certain substance use.

**Figure 3:**
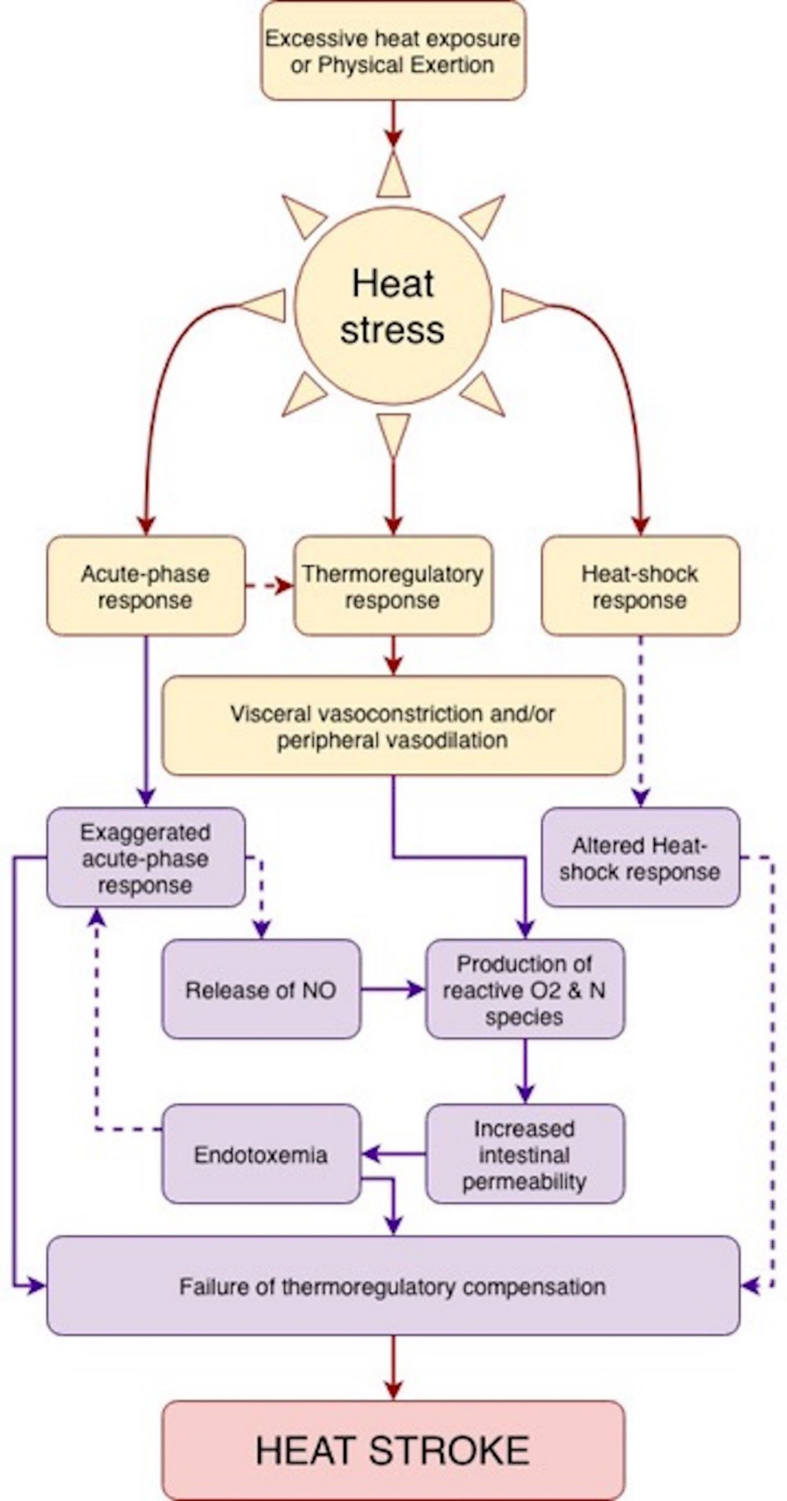
Proposed pathophysiologic sequence of the development of heat stroke. Adapted from Bouchama and Knochel [1].

Heat stroke has a complex pathophysiology, it has been postulated that body heat is gained from the environment and is also produced by its own metabolism. This overall heat load must be dissipated to maintain a body temperature of 37°C [1]. It has been observed that at extreme temperature (49°C-50°C) all cellular structures are destroyed and cellular necrosis occurs in less than five minutes [11]. The acute phase response to heat stress is a coordinated reaction that involves endothelial cells, leukocytes and epithelial cells which should lead an increased cardiac output dissipating heated blood to the periphery via vasodilation [12, 13]. Heat stroke and its progression to MODS are due to a complex back-and-forth among the acute physiological alterations associated with hyperthermia (circulatory failure, hypoxia and increase metabolic demand) and the direct cytotoxicity of heat and the inflammatory and coagulation response of the host [1]. This complex interplay has been demonstrated in Figure 3. It has also been reported that genetic factors affecting the thermoregulatory cascade such as variations in the RYR1 gene may also play a role in the susceptibility to heat stroke. The genes included are those related with cytokines, coagulation cascade proteins and proteins involved in the adaptation to heat stress [14].

Heat stroke presents with the classical triad of rectal temperature greater than 40°C, altered mental status and hot dry skin [5]. A hallmark symptom is CNS dysfunction that manifests as mental status changes, including confusion, delirium, combativeness, seizure or coma at the time of collapse, as observed in our case. Despite rapid cooling approximately 30% of heat stroke survivors experience permanent neurological impairment that may be related to cerebellar atrophy and infarcts [7, 15]. Acute renal failure is also a common finding that is exacerbated by rhabdomyolysis as was seen in our patient. While liver failure is also a recognized complication, the damage may not peak until 24-48 hours after heat insult [6]. Cardiac abnormalities include ST elevation, diffuse nonspecific ST-T change, conduction defects, prolonged QT interval, segmental or diffuse wall motion abnormalities and rarely pericardial effusion [16]. Metabolically, acidosis is most common which contributes to multiorgan dysfunction and morbidity as well.

The mainstay of treatment is immediate cooling as clinical outcomes are dependent on duration of hyperthermia [1, 17]. Methods of cooling include ice packing, immersion, evaporative cooling and invasive methods like gastric, peritoneal and bladder lavage. Agents such as dantrolene have also been used due to its ability to reduce muscle excitation and contraction [17]. Other proposed agents include recombinant human-activated protein C (rhAPC) with its anti-inflammatory and anticoagulant properties [18]. Clinical outcomes, risk of multi-organ dysfunction and overall prognosis may be estimated using chemistries such as creatine kinase, transaminases and the presence of metabolic acidosis at admission predict the development of multi-organ dysfunction [19]. One prospective study conducted during the French heat wave of 2003 demonstrated that the occurrence of at least one organ failure associated with neurological dysfunction is associated with a lower probability of survival [15].

The most serious complications of heat stroke are those falling within the category of MODS [1]. Inflammatory cytokines involved in the response of heat and strenuous exercise such as IL-1 and IL-6 modulate subsequent cytokines like TNF-α implicated in MODS. Endothelial cell injury and microvascular thrombosis are also prominent features of heat stroke [1, 20]. This in turn may lead to widespread activation of the coagulation cascade leading to microvascular thrombosis and possible disseminated intravascular coagulation.

## Conclusions

Heat stroke is an uncommon entity, especially in a younger population. Its presentation is often abrupt in the setting of physical exertion or excessive climatic heat. It may present non-specifically but often has a neurological component which may be transient or permanent in the setting of prolonged hyperthermia. Majority of cases are managed with emergent cooling to prevent lasting neurologic deficits and development of MODS. We demonstrate a clinically challenging case of heat stroke in an otherwise healthy 23-year-old male illustrating how a rapid assessment and identification accompanied with a proper management aided in a successful recovery. Clinicians should consider heat stroke in their differential when patients present with altered mental status and history suspicious of excessive heat exposure or physical exposure.

## References

[1] Bouchama A, Knochel JP (2002). Heat stroke. N Engl J Med.

[2] Royburt M, Epstein Y, Solomon Z, Shemer J (1993). Long-term psychological and physiological effects of heat stroke. Physiol Behav.

[3] Leon LR, Bouchama A (2015). Heat stroke. Compr Physiol.

[4] Mørch SS, Andersen JDH, Bestle MH (2017). Heat stroke: a medical emergency appearing in new regions. Case Rep Crit Care.

[5] Nadesan K, Kumari C, Afiq M (2017). Dancing to death: a case of heat stroke. J Forensic Leg Med.

[6] Bouchama A, Dehbi M, Mohamed G, Matthies F, Shoukri M, Menne B (2007). Prognostic factors in heat wave-related deaths: a meta-analysis. Arch Intern Med.

[7] Dematte JE, O'Mara K, Buescher J (1998). Near-fatal heat stroke during the 1995 heat wave in Chicago. Ann Intern Med.

[8] Semenza JC, Rubin CH, Falter KH (1996). Heat-related deaths during the July 1995 heat wave in Chicago. N Engl J Med.

[9] Jones TS, Liang AP, Kilbourne EM (1982). Morbidity and mortality associated with the July 1980 heat wave in St Louis and Kansas City, Mo. JAMA.

[10] Berko J, Ingram DD, Saha S, Parker JD (2014). Deaths attributed to heat, cold, and other weather events in the United States, 2006-2010. Natl Health Stat Report.

[11] Buckley IK (1972). A light and electron microscopic study of thermally injured cultured cells. Lab Invest.

[12] Gabay C, Kushner I (1999). Acute-phase proteins and other systemic responses to inflammation. N Engl J Med.

[13] Rowell LB (1983). Cardiovascular aspects of human thermoregulation. Circ Res.

[14] Moseley PL (1997). Heat shock proteins and heat adaptation of the whole organism. J Appl Physiol.

[15] Argaud L, Ferry T, Le QH (2007). Short- and long-term outcomes of heatstroke following the 2003 heat wave in Lyon, France. Arch Intern Med.

[16] Akhtar MJ, Al-Nozha M, Al-Harthi S, Nouh MS (1993). Electrocardiographic abnormalities in patients with heat stroke. Chest.

[17] Smith JE (2005). Cooling methods used in the treatment of exertional heat illness. Br J Sports Med.

[18] Bouchama A, Kunzelmann C, Dehbi M (2008). Recombinant activated protein C attenuates endothelial injury and inhibits procoagulant microparticles release in baboon heatstroke. Arterioscler Thromb Vasc Biol.

[19] Varghese GM, John G, Thomas K, Abraham OC, Mathai D (2005). Predictors of multi-organ dysfunction in heatstroke. Emerg Med J.

[20] Leon LR, Helwig BG (2010). Heat stroke: role of the systemic inflammatory response. J Appl Physiol.

